# Forest Density Drives Survival and Trait Variation in South European Understorey Species: A Continental‐Scale Translocation Experiment

**DOI:** 10.1111/ele.70184

**Published:** 2025-08-04

**Authors:** Cristina Gasperini, Elisa Carrari, Karen De Pauw, Giovanni Iacopetti, Sofia Martini, Pieter Sanczuk, Thomas Vanneste, Pieter Vangansbeke, Pieter De Frenne, Federico Selvi

**Affiliations:** ^1^ Department of Agriculture, Food, Environment and Forestry University of Florence Florence Italy; ^2^ Forest & Nature Lab, Department of Environment, Faculty of Bioscience Engineering Ghent University Ghent Belgium

**Keywords:** altitudinal translocation, flowering, forest density, forest edge, functional traits, intraspecific variability, latitudinal translocation, Mediterranean understorey species, microclimate, plant survival

## Abstract

Despite their importance for forest biodiversity and functioning, little is known about the responses of south European understory herbs to climate change. We used a translocation experiment in southern and central Europe to unravel the short‐term effects of macroclimatic (elevation and latitude) and microclimatic conditions (open vs. dense forests, forest edge vs. core position) on plant survival, flowering and traits in eight understorey specialists. Forest density was the main driver of survival, with positive effects in the warm and water‐limited southern region and negative effects in the northern oceanic region. Forest position had weaker effects, influencing survival, growth and SLA in contrasting ways at the two latitudes. Most species flowered beyond their northern limit, suggesting the ability for reproduction at higher latitudes. Macroclimate effects on forest herbs interact with forest density, influencing their performance and suggesting complex responses to climate change. Increased vulnerability is expected in relatively open and warmer forests.

## Graphical abstract

Using a multifactorial plant translocation experiment along elevation, latitude, forest density (dense vs. open) and position (edge vs. core), we tested survival, flowering and growth‐related traits in eight understorey species from S Europe. Forest density was the main driver of plant survival, with positive effects in Italy and negative in Belgium. Most species could flower beyond their northern range limit.

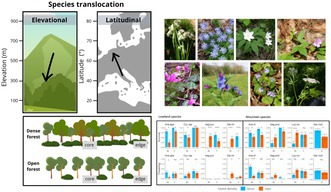

## 1 Introduction

Understorey species represent most of the plant diversity in temperate forests and are often vulnerable to environmental changes because of their narrow ecological niches and limited dispersal capacities (Ehrlén and Eriksson 2000; Landuyt et al. 2019). Increasing pressures driven by land use changes, forest fragmentation, silvicultural management and extreme climate events can significantly reduce the population size and distribution range of many specialist herbaceous taxa of the understorey (Chelli et al. 2024; Økland et al. 2023; Wen et al. 2022). Mountain species appear particularly vulnerable to climate change because of their sensitivity to warming and limited chances for altitudinal migration (Rogora et al. 2018). However, lowland species are also subject to pressures and threats owing to their distribution across highly fragmented forest landscapes, hence with reduced opportunities for dispersal to suitable habitat (Bertrand et al. 2011). Whether understorey species will actually be able to shift their range for tracking their climatic envelope is unlikely in most cases (Lenoir and Svenning 2015). In view of these limitations, it is crucial to unveil how these species can respond to rising temperatures through phenotypic and phenological adaptation, considering that increases in temperature associated with anthropogenic climate change are rapidly driving many of them towards their upper thermal limits (Adams et al. 2009; Sanczuk et al. 2023; Wei et al. 2024). At high temperatures, such as those increasingly occurring in southern Europe, physiological processes such as photosynthesis, transpiration, water transport and growth could suffer a significant reduction, even if biotic interactions are favourable (Bussotti et al. 2014; Zhao et al. 2020). In southern Europe, understorey species of mountain beech forests with cool climates are less tolerant to thermal and water stress than those of lowland thermophilous forests because of poor control of transpiration, which may lead to a decline in their survival and growth with rising temperatures and increasing drought (Pignatti 1998).

Manipulative species translocation experiments are an effective approach to examine phenotypic and functional responses to future conditions. Latitudinal translocation of central and northern European species close to the southern and/or northern limit has been performed to examine plant traits and phenological patterns in different climatic contexts (De Frenne et al. 2011; De Pauw et al. 2022; Van der Veken et al. 2012). However, translocation of southern European understorey herbs beyond their northern limit has not been performed so far, though this kind of experiment can help predictions about species performances at higher latitudes where they could spread in the future because of isotherm tracking (Hargreaves et al. 2014). It is actually unknown whether the inherently different macroclimatic features of a European oceanic region in terms of seasonal patterns of atmospheric circulation and humidity, precipitation and light regimes may influence survival, flowering and traits in these species. Similarly, no altitudinal translocation experiments of mountain species to warmer lowland forests in southern Europe have been carried out at present, despite their potential to bring light on responses to higher temperatures expected in the next decades in mountain areas of this region.

Translocation along latitudinal or elevational gradients involves changing macroclimatic conditions, but microclimate also plays a key role in modulating responses of understorey species (De Frenne et al. 2019). Forest structure can either mitigate or exacerbate the effects of heat and drought on understorey plants, as microclimatic buffering depends on cover and density of the tree and shrub layers (Chelli et al. 2024; Sanczuk et al. 2023). The stronger the reduction of density and cover because of management, the stronger the reduction in its temperature buffering capacity (Meeussen et al. 2021; Santi et al. 2024). Microclimatic conditions also vary with distance to forest edges, with decreasing buffering capacity from the core to the edge. Moreover, forest density influences this edge‐to‐core pattern with a deeper edge influence under a reduced stand density (Meeussen et al. 2021). Increased light availability, larger temperature extremes and altered wind circulation that usually characterise forest edges translate into direct and indirect effects on plant species and communities, overall defined as “edge effect” (Vanneste et al. 2024).

Functional traits are increasingly recognised as predictors of plant responses to climate change, in terms of abiotic stress tolerance (e.g., Sanczuk et al. 2023; Wei et al. 2024) and biotic interactions such as competition (Lyu and Alexander 2024). Besides survival, in this study, we focused on flowering, plant height, leaf number and specific leaf area (SLA), all of which are proxies for different aspects of plant function and fitness. Flowering represents a critical life‐history trait indicating reproductive success and the potential for population persistence and range expansion (Roeber et al. 2022), whereas plant height and leaf number are linked to competitive ability, growth potential and resource acquisition (Westoby et al. 2002). SLA is an indicator of a species’ resource‐use strategy and its ability to balance light capture with water conservation (Poorter et al. 2009; Wright et al. 2004). It mostly reflects a trade‐off between photosynthesis and water conservation, often influenced by light and water availability (Burton et al. 2017; Poorter et al. 2019). Intraspecific variation in this trait runs counter to expectations associated with the Leaf Economic Spectrum on the basis of interspecific differences, according to which SLA increases in resource‐acquisitive species with high photosynthetic capacity (Burton et al. 2020). Within species, in fact, increasing light usually decreases SLA as a result of enhanced tissue structural investments and cell thickness (Givnish 1988). Recent evidence supports that microclimatic conditions of open forests and edges have contrasting effects on this trait depending on the bioclimatic region and the species (De Pauw et al. 2022). Although a higher SLA may optimise light capture in cool and moist habitats, a lower SLA is likely an adjustment to minimise transpiration in water‐limited environments. This was recently supported by a study on understorey herbs from southern European oak forests subject to summer drought. These species decreased their SLA when growing in open stands because of coppicing (Santi et al. 2025). Yet, the patterns of intraspecific variability and survival in understorey species of the Mediterranean region in relation to macroclimatic and microclimatic conditions are still largely unknown, though this region is globally one of the most affected by climate change (Balzan et al. 2020; Cuttelod et al. 2008; Peñuelas and Sardans 2021).

Accordingly, we set up a plant translocation experiment along four gradients: (1) elevation (mountain to lowland), (2) latitude (Italy to Belgium), (3) forest density (dense vs. open canopies), and (4) position (forest core vs. forest edge). This multifactorial design allowed us to test the effect of macroclimate and microclimatic changes on survival, flowering and traits (plant height, cover, SLA and leaf number) in eight south European understorey species of mountain beech and lowland oak forests. We hypothesised that: (i) forest density and position have a significant influence on species survival, flowering and growth, with the latter assessed through metrics such as plant cover, height and leaf number, (ii) the direction and magnitude of species responses to microclimatic variation are different within their native southern range or mostly beyond their northern limit. Specifically, we expected higher survival, flowering and growth in dense forests and core position, where microclimatic buffering is stronger, particularly in the warmer and drier southern region. In contrast, open forests and edge position may have more positive effects in the cooler northern region, thanks to increased light availability and warmer microclimate favouring growth and flowering, and (iii) mountain and lowland species respond differently, the former showing stronger reduction in survival and growth when translocated to warmer and drier lowland forests and the latter exhibiting a broader tolerance under similar conditions.

## 2 Material and Methods

### 2.1 Study Species

The eight understorey species investigated in this study are presented in Table 1, briefly described and illustrated in Text A1, Table A1, Figures A1 and A2. The plant material used for the experiment is also described in Text A1.

**TABLE 1:** Summary of the study species and of the relative sites of sampling, showing phylogenetic relationships, flowering period and forest guild, after Heinken et al. (2022; 1.1: Forest specialist; 2.1: Forest generalist). Mean Annual Temperature (MAT) and Mean Annual Precipitation (MAP) were derived from Worldclim2 (30‐year average from 1970 to 2000, resolution of 2.5 arcminutes; Fick and Hijmans 2017). Raunkiaer’s life form, chorotype and Ellenberg indicator values of the species are shown in Table A3. Species nomenclature follows Euro+Med (2006).

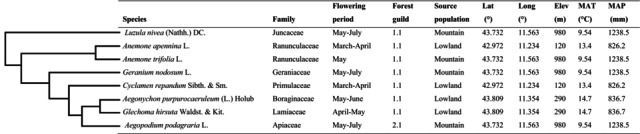

### 2.2 Study Sites and Experimental Design

**FIGURE 1:**
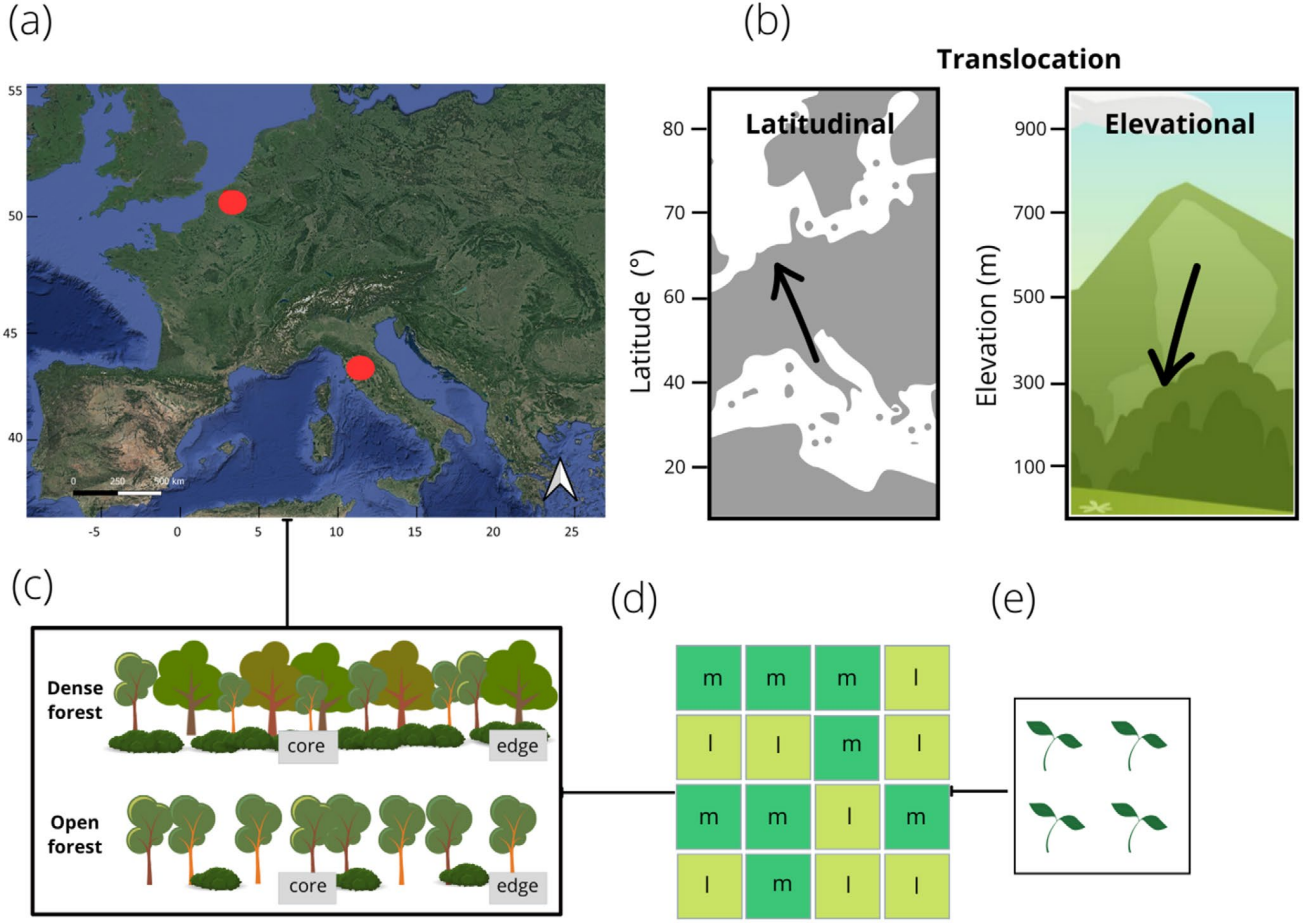
Study design showing: (a) experimental sites in Belgium and Italy; (b) latitudinal and altitudinal directions of translocation underlying macroclimatic gradients; elevational translocation was only performed in Italy; (c) structural types (open and dense) and positions (forest edge and forest core) underlying microclimatic gradients; (d) plot with 16 boxes, two boxes per species (replicates), with randomly distributed mountain species (m, dark green) and lowland species (l, light green); (e) single box with four individuals of one species. Dark green represents the boxes with the mountain species, light green those with the lowland species (l) (see also Figures A3–A5).

The experiment was conducted in two distant European regions, central Italy (43.67° N, 11.14° E) and northwest Belgium (50.97° N, 3.80° E), with a sub‐mediterranean and oceanic climate type, respectively (Beck et al. 2018; Figure 1a). In both regions, we installed four plots at low‐altitude sites dominated by *Quercus robur* in Belgium and by *Q. pubescens* and *Q. cerris* in Italy. The sites represented two forest density levels, for example, dense (multilayered tree canopy and shrub layer with ground cover > 80%, basal area 20–40 m^2^) and open (single tree layer with canopy cover < 70%, basal area 8–24 m^2^, shrub layer cover < 45%). At each site and density level, one plot was installed at the forest edge (ca. 2 m from the outermost tree trunks; Meeussen et al. 2021) and one at the forest core (ca. 100 m from the forest edge; Figure 1). More site details are given in Table A2 and a scheme of the experimental design is shown in Figure A3.

In February–April 2021, 1‐year old plants of the eight study species were brought to the transplanting sites, before the start of the growing season. In each plot, 16 boxes (two replicates for each species) were randomly installed side by side (Figure 1d). Four individuals of one out of eight species were placed in each box, thus excluding interspecific competition from the experiment. Two boxes for each species per location were installed (Figure 1e).

Plants were therefore translocated northwards to ca. 1300 km from their native sites (Figure 1b), mostly beyond the northern limits of the species range (Figure A2). Mountain species were also translocated to lowland sites in Italy, along a descending gradient of ca. 800 m (Figure 1b). The differences in the mean annual temperature (MAT) between the site of plant collection (on the basis of data from Worldclim2 [Fick and Hijmans 2017]) and the sites where plants were translocated (local temperature data collected with TMS‐4 loggers; TOMST, Prague, Czech Republic) are shown in Table A3. Measurements of photosynthetic active radiation (PAR) were taken at 35 cm from the forest floor with a PAR Quantum sensor SKP 215 (Skye Instruments Ltd., Llandrindod Wells, UK) on 1 day in Italy and Belgium during the full growing season (July), during midday conditions (12:00 h–14:00 h) with clean sky; values are given in Table A4.

### 2.3 Field Data Collection

Data were collected at three time points corresponding to the peak of vegetative growth and flowering period of the species, for example, April for lowland species, the end of May and mid of July for mountain species. Data collection was repeated in 2022, at the same three time points. At each survey, the following data were recorded: (i) survival (number of living plants), (ii) number of flowering plants, (iii) percentage of individual plant cover (visual estimation on the basis of a grid with 50 × 50 mm meshes laid down in the boxes), (iv) number of leaves per individual and (v) natural plant height, that is, without stretching the plant and not considering the flowering stalk (Pérez‐Harguindeguy et al. 2013). Next, one well‐developed leaf from two healthy individuals per species per box was collected. The leaf area (LA) was determined with an LI‐3100C Area Meter (Li‐Cor Environmental, Lincoln, NE, USA). Leaves were then dried in an oven at 40°C for 48 h and weighed (to 0.1 mg).

### 2.4 Data Analysis

We used data collected in April for the early‐spring flowering species (*C. repandum, A. apennina* and *A. trifolia* ) and data collected in May and July for the spring and summer flowering species (*A. purpurocaeruleum, G. hirsuta, A. podagraria, L. nivea* and *G. nodosum*, the latter only in Italy) for two consecutive years, 2021 and 2022.

We fitted generalised linear mixed effects models (GLMm and LMMs) with measurement data for all translocated individuals (ca. 1500 observations), grouped by elevational provenance (mountain vs. lowland species) and separately for each species (> 124 obs. for lowland species, > 240 obs. for mountain species) using the packages'lme4’ (Bates et al. 2015) and ‘MuMln’ (Bartoń 2019). Plant responses to translocation were analysed in relation to a set of explanatory variables, namely region (Italy vs. Belgium), forest density (dense vs. open) and plot position (edge vs. core). The responses to the elevational translocation were separately addressed for the mountain species. Mean values of the response variables are given in Tables B2–B4, separately per group (mountain vs. lowland) or species. All variables were scaled before the analysis, except binomial ones. A binomial distribution was used for survival and flowering data, a Poisson distribution for the number of leaves, whereas a Gaussian distribution was applied for the other response variables. All fixed effects were categorical variables, and all two‐way interactions were considered. In the models with grouped response variables (Equation 1) and divided for mountain vs. lowland species, the “year” was included as a random effect and the “species/plantID” as a nested random effect to account for repeated measurements and interspecific variability, respectively. In models separated by species (Equation 2), “year” and “plantID” were added as random intercept terms to account for repeated measurements.

The following formulas represent the starting models:

(1) $$\begin{array}{c} \text{Variable}\sim {\left(\text{Region}+\text{Forest density}+\text{Edge}\;\mathrm{vs}.\;\text{Core}\right)}^{2}\\ +\left(1\;|\;\text{year}\right)+\left(1\;|\;\text{species}/\text{plantID}\right) \end{array}$$

(2) $$\begin{array}{c} \text{Variable}\sim {\left(\text{Region}+\text{Forest density}+\text{Edge}\;\mathrm{vs}.\;\text{Core}\right)}^{2}+\left(1\;|\;\text{year}\right)\\ +\left(1\;|\;\text{plantID}\right) \end{array}$$

In an additional model, the two‐factor variable “shade tolerance” was included in Equation (1) to evaluate its effects on survival, flowering, and growth responses in the different experimental conditions. Species were grouped based on Ellenberg indicator values for light (3–4: higher shade tolerance; 5–6: lower shade tolerance; Text A1).

The lowest Akaike information criterion (AIC) and Bayesian information criterion (BIC) were used to select the best‐fitting model through an automated model selection process, using the dredge function from the MuMIn package (Bartoń 2019; Fieberg and Johnson 2015), which ranks all possible subsets of the global model. In all models, we visually checked for model assumptions (homogeneity of variance, normality of random effects and normality of residuals). Multicollinearity of the predictor variables in the models was assessed using variance inflation factors and was not present (VIFs < 5) (Zuur et al. 2009). Since the limited number of observations did not allow the use of a negative binomial distribution when overdispersion occurred, we adjusted the coefficient table by multiplying the standard error by the square root of the dispersion factor and recomputing the *z*‐ and *p*‐values accordingly (Lee and Nelder 2000).

For all models, the proportion of variance explained by the fixed effects (marginal *R* ^2^) and by both random and fixed effects (conditional *R* ^2^) was computed (Nakagawa and Schielzeth 2013). A post hoc test (Tukey multiple comparisons) was performed with the function TukeyHSD (package stats) to assess significant interactions. All analyses were performed in R v. 4.1.0 (R Core Team 2022).

## 3 Results

### 3.1 Plant Survival and Flowering

**FIGURE 2:**
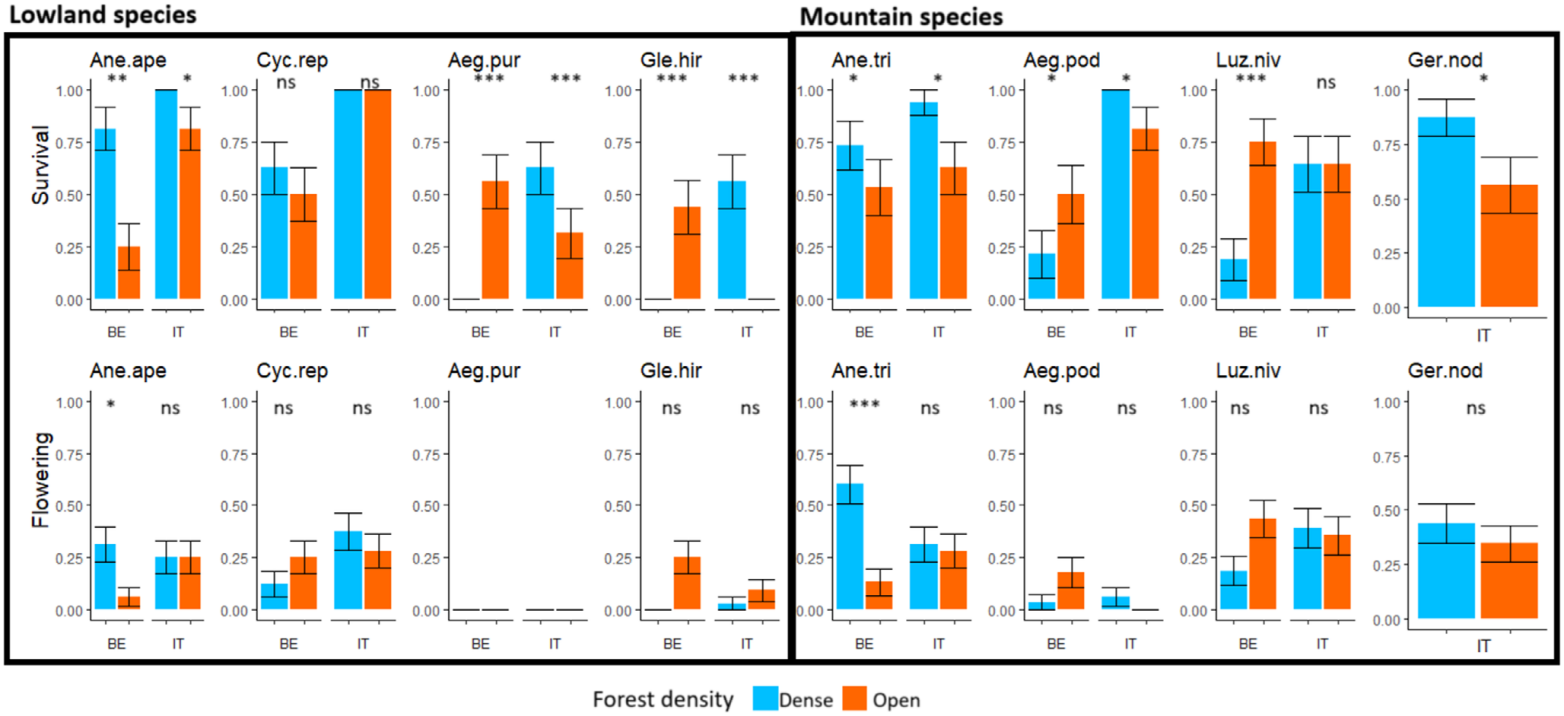
Plant survival (at the end of the second year, scored as 0 when the plant died, and 1 when the plant was alive) and flowering probability (scored as 1 when the plant flowered or as 0 when not, throughout the experiment) grouped by region (Italy vs. Belgium) and forest density (dense vs. open). Significant interactions between region and forest density (on the basis of lmer and glmer) are shown for each species: Ane.ape = *Anemone apennina*, Cyc.rep = *Cyclamen repandum* , Aeg.pur = *Aegonychon purpurocaeruleum*, Gle.hir = *Glechoma hirsuta*, Ane.tri = *Anemone trifolia* , Aeg.pod = *Aegopodium podagraria* , Luz.niv = *Luzula nivea* , Ger.nod = *Geranium nodosum*. A post hoc Tukey test was performed to assess significant differences between forest densities within each region (*p* < 0.05*, *p* < 0.01**, *p* < 0.001***). *Geranium nodosum* was only translocated within Italy (see Section 2.3).

Plant survival after translocation was significantly higher in Italy (72%) than in Belgium (44%), without significant differences between mountain and lowland species (Table 2 and Table B2). In both groups, the open forest structure had a negative effect in Italy (60% vs. 83% in dense forests) but was positive in Belgium (52% vs. 37%; Table B3). Survival of mountain species was higher at the forest core than at the edge in both regions (Table B4). However, responses were in some cases species‐specific and dependent on the region. The open forest increased mortality in *A. apennina* and *A. trifolia* in both regions, as well as in *G. nodosum, A. purpurocaeruleum*, *G. hirsuta* and *A. podagraria* in Italy (Figure 2). In Belgium, there was an opposite (positive) effect on the survival of the latter three herbs and the graminoid *L. nivea* (Figure 2).

**TABLE 2:** Summary table of significant effects of lmer and glmer determined by region (Italy vs. Belgium), forest density (dense vs. open) and edge (vs core) position on understorey species groups (four mountain and four lowland species) in terms of survival, reproductive performance, growth, and functional traits. Direction of the arrows indicates positive (upward) or negative effects of the independent variable on the dependent variable. Edge position, Belgium and dense forest are used as reference categories. Asterisks denote significance levels, with *p* < 0.05*, *p* < 0.01**, *p* < 0.001***. Marginal *R* ^2^ and conditional *R* ^2^ (Nakagawa and Schielzeth 2013) represent the proportion of variation explained by fixed factors and both random and fixed factors, respectively.

Variable	Group	Italy (vs. Belgium)	Open forest (vs. Dense)	Core position (vs. Edge)	Open forest X Italy	Core position X Italy	Core position X Open forest	*R* ^2^ marginal	*R* ^2^ conditional
Survival	Mountain	↑***	↑***	↑**	↓***		↓***	0.18	0.71
	Lowland	↑***	↑***	↓	↓***	↑**	↓***	0.15	0.58
Flowering	Mountain			↑				0.004	0.12
	Lowland		↑**	↑			↓***	0.03	0.52
Plant cover	Mountain	↑	↑		↓***			0.02	0.21
	Lowland	↑	↑***	↓	↓***		↓**	0.09	0.21
Plant height	Mountain	↓	↑***	↑	↓**	↑*	↓**	0.07	0.50
	Lowland	↑**	↑***	↑	↓**		↓**	0.04	0.34
Leaf number	Mountain	↓***	↑***	↓	↓***	↑***	↓**	0.12	0.97
	Lowland	↓	↑***	↓	↓	↓	↓***	0.02	0.97
SLA	Mountain	↑**	↓	↑***		↓**	↓	0.06	0.59
	Lowland	↑**	↓***					0.08	0.33

**FIGURE 3:**
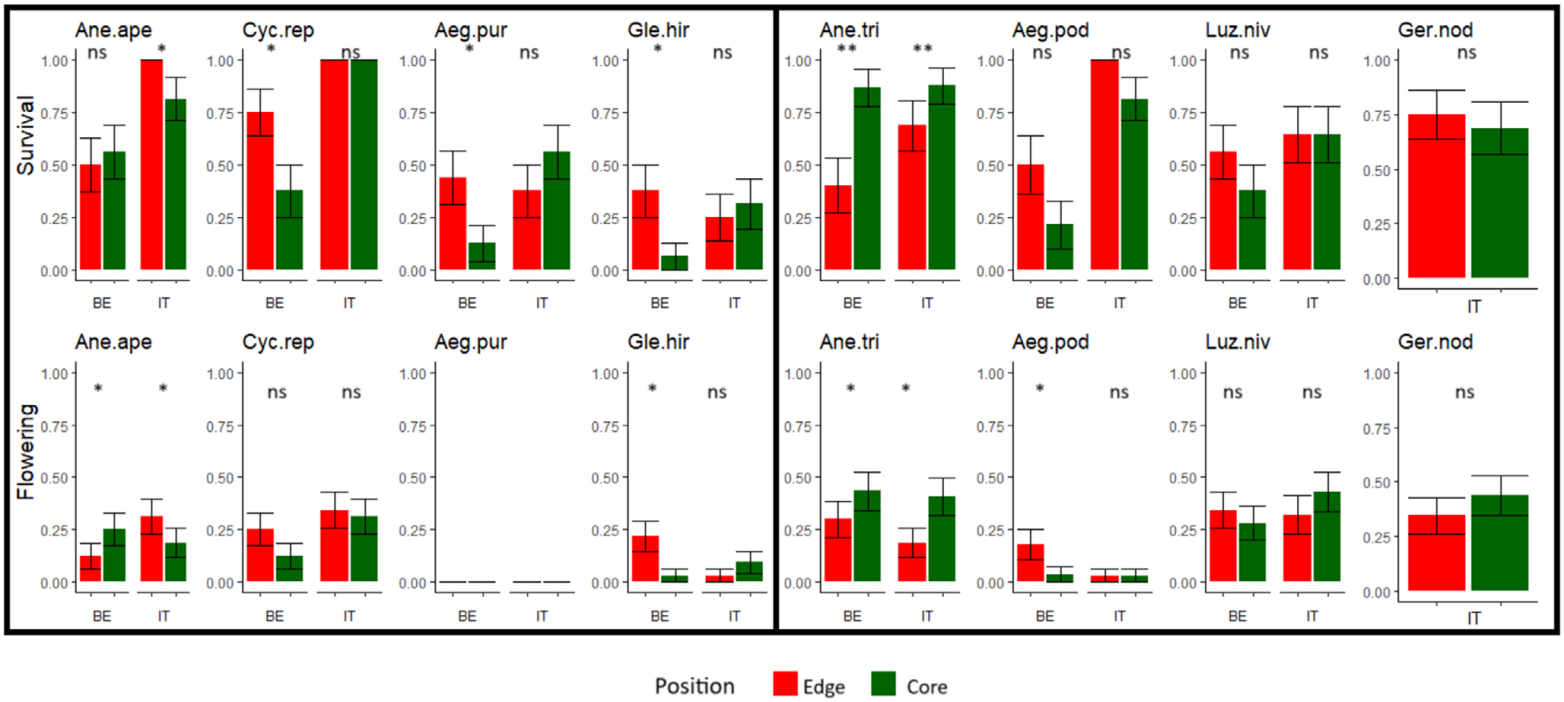
Plant survival (at the end of the second year, scored as 0 when the plant died, and 1 when the plant was alive) and flowering probability (scored as 1 when the plant flowered or as 0 when not, throughout the experiment) grouped by region and forest edge vs. core. Significant interactions between region and forest density (on the basis of lmer and glmer) are shown for each species: Ane.ape = *Anemone apennina*, Cyc.rep = *Cyclamen repandum* , Aeg.pur = *Aegonychon purpurocaeruleum*, Gle.hir = *Glechoma hirsuta*, Ane.tri = *Anemone trifolia* , Aeg.pod = *Aegopodium podagraria* , Luz. niv = *Luzula nivea* , Ger. nod = *Geranium nodosum*. A post hoc Tukey test was performed to assess significant differences between edge vs. core within each region, denoted by asterisks (with *p* < 0.05*, *p* < 0.01**).

Edge versus core position influenced the survival of most species in Belgium (4 out of 7), but only in two species in Italy (Figure 3). In Belgium, *C. repandum* , *A. purpurocaeruleum* and *G. hirsuta* survived more at the forest edge, whereas no differences occurred in Italy (Figure 3). Mortality of *A. trifolia* was instead higher in the edge position in both regions. Lowland species survived more at the forest core in Italy, but not in Belgium (significant interaction Core position × Italy, Table 2), whereas no effects were detected for mountain species. The core position in open forests negatively influenced species survival in either mountain or lowland species in both regions (Table 2).

All species reached sexual maturity in both countries, with the only exception of *A. purpurocaeruleum*, which always remained in a vegetative phase. The proportion of flowering individuals ranged from 10% in *A. podagraria* to 44% in *C. repandum* . Flowering of lowland species was promoted in open forests, whereas mountain species were not affected by forest density (Table 2). In Belgium, the proportion of flowering individuals of *A. apennina* and *A. trifolia* was significantly higher in the dense forest, whereas *G. hirsuta* performed better in the open stands (Figure 2). In Belgium, *C. repandum* and *G. hirsuta* flowered more at the edge than at the core (Figure 3). By contrast, flowering of *A. trifolia* in both regions was higher at the forest core than at the edge (Figure 3).

### 3.2 Plant Traits

**FIGURE 4:**
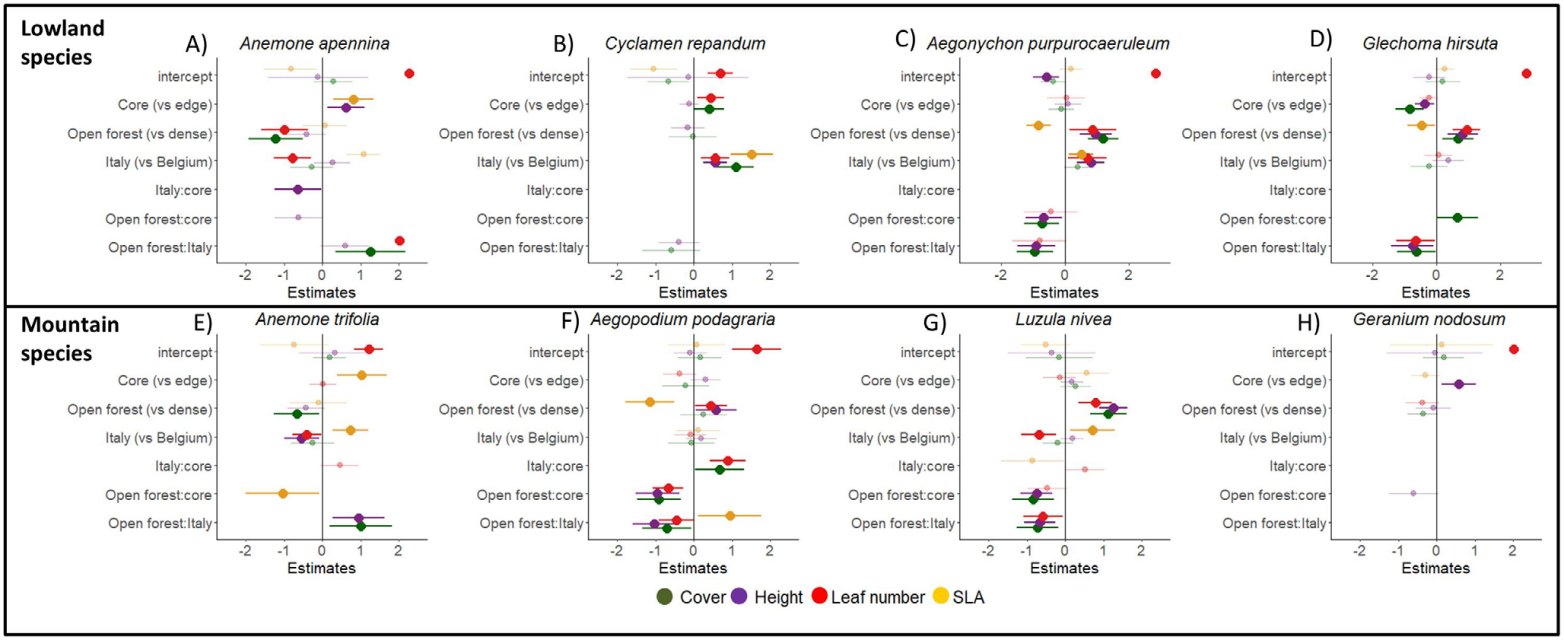
Estimates and 95% confidence intervals for the explanatory variables: Edge vs. core, forest density and region. Edge position, dense forest and Belgium were used as reference categories for the comparison, respectively. Significant variables are in bold. Variables or interactions that are not shown were not included in any final model (lmer and glmer). *Geranium nodosum* was only investigated in Italy. Marginal and conditional *R* ^2^ of the models are given in Table B1. Lowland species: A) Anemone apennina, B) Cyclamen repandum, C) Aegonychon purpurocaeruleum, D) Glechoma hirsuta; Mountain species: E) Anemone trifolia, F) Aegopodium podagraria, G) Luzula nivea, H) Geranium nodosum.

In Italy, open forests had a negative influence on the ground cover of both mountain and lowland species, particularly in *C. repandum, A. podagraria* and *G. nodosum* (Table 2, Figure 4 and Figure B1). In Belgium, the effect on plant cover was instead generally positive, particularly in *A. purpurocaeruleum, G. hirsuta* and *L. nivea* (Table 2; Figure 4 and Figure B1), although *A. apennina* and *A. trifolia* showed an opposite response (higher cover in the dense forests; Figure 4 and Figure B1). The mean cover of the geophyte *C. repandum* was always higher at the forest core, whereas this position had a negative effect on *G. hirsuta* (Figure 4).

Open forests in Belgium had an overall positive influence on the plant height of both mountain and lowland species (Table 2), especially in *A. purpureocaeruleum, G. hirsuta, A. podagraria* and *L. nivea* (Figure B1). This was not observed in Italy, where a negative response was shown by *A. podagraria* (Figure B1). Lowland species grew significantly taller in Italy than in Belgium (Table 2), in particular *C. repandum* and *A. purpureocaeruleum* (Figure 4). Also, mountain species were significantly taller at the forest core than at the edge (Table 2). The core position enhanced plant height in *G. nodosum* and *A. apennina*, whereas it had a negative effect on *G. hirsuta* .

Forest density also influenced the number of leaves. If there was a significant difference in the number of leaves between regions, then the plants had significantly more leaves in Belgian open forests than in the Italian ones (Table 2). However, this did not happen consistently in both regions for all species (Figure B1). The core position increased the leaf number of mountain species only in the dense forest, whereas in open forests, there was a negative effect (Table 2, interaction terms). Only *C. repandum* showed a significant response when analysing species separately, with a higher leaf number at the forest core (Figure 4). The leaf number in mountain species ( *A. trifolia* and *L. nivea* ) was on average higher in Belgium (Table 1), whereas for the two lowland species, *C. repandum* and *A. purpureocaeruleum*, it was higher in Italy (Figure 4).

The specific leaf area of both mountain and lowland species was generally higher in Italy than in Belgium (Table 2), especially in *C. repandum* , *A. purpureocaeruleum, A. trifolia* and *L. nivea* (Figure 4). In lowland species, SLA decreased in the open forest of both regions (Table 2), particularly in *A. purpureocaeruleum* and *G. hirsuta* (Figure 4, Figure B1), whereas a similar effect was observed in *A. podagraria* in Belgium (Figure B1). The edge position reduced SLA in mountain species in Belgium, whereas an opposite effect was observed in Italy (Table 2). At the species level, SLA was significantly affected by position only in *A. trifolia*, being lower at the core of open forests (Figure 4, Table B2). More shade‐tolerant species exhibited significantly higher flowering than less shade‐tolerant species, both at forest edges and cores (Figure B2). Conversely, less shade‐tolerant species achieved greater ground cover than more shade‐tolerant species at the edge, but not at the core. No significant differences between the two groups were observed for leaf number across regions, nor SLA in relation to forest density, although these interactions were significant in GLMm and LMM models.

## 4 Discussion

This study examined for the first time the responses of southern European understorey species from Mediterranean mountain beech and lowland oak forests to changes in microclimatic conditions at the forest edge versus core and in dense versus open forests and to macroclimatic changes related to elevation and latitude. Overall, our findings support forest density and, to a lesser extent, edge versus core position as drivers of species survival and performance, with different effects depending on the region. Unexpectedly, the elevational translocation of mountain species to lowland forest resulted in responses largely in line with those of lowland species. We first hypothesised that forest density and position (edge vs. core) have significant effects on species survival, flowering, and growth, and, secondly, that responses to microclimatic variation are different in their native southern range and beyond their northern limit (i.e., in Belgium).

Forest density resulted as the main variable driving the survival and growth of the study species, though the direction of the effect depended on the region as expected from our first two hypotheses. In their area of origin (C Italy), species performance was generally worse in the open plots, because of the weakened microclimatic shelter of stands with reduced tree stock and canopy cover (Zellweger et al. 2019). More intense solar radiation, higher temperatures and associated levels of water stress were likely causes for the higher plant mortality in the open plots during our 2‐year experiment. In recent years, forest plant survival and growth has declined in Italy (Bussotti et al. 2014; De Frenne 2023; Iacopetti et al. 2021; Ogaya and Peñuelas 2021), where daily maximum temperatures have continuously exceeded the 30‐year record for that day (Arcidiaco et al. 2024). Forest density is thus confirmed to play a key role in driving the responses and population dynamics of understorey plants under current global warming (Govaert et al. 2020; Lelli et al. 2019; Sanczuk et al. 2023) Recent evidence suggests that when canopy cover drops below *c*. 75%, the degree to which forest understoreys will be buffered from extremes in temperature and vapour pressure deficit is severely reduced (De Frenne et al. 2021; Zellweger et al. 2019). Moreover, the influence of forest structure on microclimate and understorey is especially strong in warm regions such as the Mediterranean (Meeussen et al. 2021), as shown by Santi et al. (2024), who recently documented significant understorey changes caused by coppicing in thermophilous deciduous forests of central Italy. Our results thus provide experimental support to previous findings from observational studies.

On the other hand, most study species performed better in the open stands when translocated to the cooler oceanic climate of northwest Belgium (Mean Annual T difference = 7°C), supporting the hypothesis that the influence of forest structure on the performance of understory plants depends on the macroclimatic context. This response is likely explained by the positive combination of higher temperatures and light in the open stands of a region where warmth and solar radiation in early spring and summer are usually less available than in central Italy (see PAR values in Table A4). Light has indeed a primary role in understorey functional responses, as shown by previous experiments where this factor enhanced growth and resulted in taller plants with higher cover (Blondeel et al. 2020; De Pauw et al. 2022; Tinya and Ódor 2016). The open structure favoured hemicryptophytes with lower shade tolerance and stronger colonising ability, such as *G. hirsuta* and *A. purpurocaeruleum*, but not the more shade tolerant geophytes *A. trifolia* and *A. apennina*, which performed better in the dense stands as in their country of origin.

Translocation to the north resulted in improved performances also in terms of flowering, with six species (out of seven) and significant individual proportions reaching the reproductive phase despite the short experiment duration. In most species, floral transition is controlled by a combination of light signals and temperature optimising seed production in specific environments (Jackson 2009; Li et al. 2016; Roeber et al. 2022). Hence, our finding shows that the combination of oceanic climatic features and light conditions at the Belgian site latitude, especially in the open stands, was still suitable to promote the induction of sexual reproduction in our southern understorey species. However, the chances for these species to migrate to the north appear quite limited and dependent on the ability to produce and disperse seeds beyond physical barriers and across the highly fragmented habitats of Europe (Loarie et al. 2009). Further studies on a wider range of understorey species in different functional types are required to better understand the degree to which our findings can be generalised.

Our third hypothesis predicted different responses in mountain and lowland species, with mountain species showing a stronger reduction in survival and growth when translocated to lowland forests compared to lowland species. Overall, we found little support for this hypothesis, since mountain and lowland species showed similar responses. Dense forests at low altitudes can therefore provide suitable refugia even for mountain species exposed to a macroclimatic warming of up to ca. 4.5°C with respect to their site of origin. Plant survival, growth, and reproduction usually decrease as local temperatures reach or exceed the warm limit of the species' thermal niche (Wei et al. 2024), showing that the limits of our mountain species were not exceeded to a harmful degree below the canopy of dense lowland oak forests. However, the ca. 1.1°C difference in the mean level of warming that these plants experienced with translocation to low altitude in the dense forest (+4.2°C) compared with the open forest (+5.3°C) may have contributed to the negative effect of the open plots. Similarly, the considerable difference in maximum temperatures between open and dense stands in our study sites (on average 6.5°C lower in the dense stands, Table A3) also explains the better performance of both mountain and lowland species in the dense forests of Italy.

Compared with forest density, position had a weaker effect on our species, and this was again region‐dependent. Overall, edge effects on plant survival and growth were stronger and positive in Belgium, supporting the beneficial influence of warmth and light on Mediterranean species growing beyond their northern range limit in an oceanic region. This is supported by the low survival of most of them (five out of seven) in the forest interior, because of cooler microclimate and lower light during the growing season. The weaker edge position effect at the Italian sites was likely due to local factors reducing microclimate effects such as local topography, edge age, tree and shrub composition and density (Govaert et al. 2020; Schmidt et al. 2017; Table A3). Although most species showed different responses to position in the two regions, the mountain geophyte *A. trifolia* exhibited a lower mortality at the forest core than at the edge in both regions. An increase in the mean cooling of ca. 0.5°C at the forest core compared to the edge may have allowed a 33% increase in its survival. Intraspecific SLA responses were driven by different design factors in lowland and mountain species. In lowland species, responses depended on forest density and consisted of a general decline in open forests, in line with evidence that within‐species SLA usually decreases with increasing light because of enhanced investment in structural tissues (Burton et al. 2017, 2020; Givnish 1988). Limited water availability, often associated with stronger solar radiation in open forests, may also contribute to intraspecific reduction in SLA, as recently found in 10 understory species of Italian coppice forests (Santi et al. 2025). In the mountain species, intraspecific SLA responses depended more on edge versus core microclimate and were region‐dependent, suggesting a different influence of macroclimatic conditions of both the elevational origin of the species (mountain vs. lowland) and the region of translocation (Mediterranean vs. oceanic). In mountain species, SLA decreased at the forest edge only in Belgium, pointing again to a negative effect of increased light availability. Negative effects on SLA were also found in understorey species of European forests growing in forest edges (Govaert et al. 2024), as a likely defence from higher transpiration rates. On the other hand, the increase of SLA in the mountain species and edge position in Italy went in an opposite direction, possibly because of local factors, such as edge structure and age or slope aspect (Govaert et al. 2021). Overall, the interactive effects of drought and light availability on SLA are complex (Burton et al. 2017), and additional information is required for a deeper understanding of the role of forest density on this trait in understorey species.

## 5 Conclusions

Adopting a novel translocation experiment along latitude and altitude, we showed that forest density is a major driver of survival, growth and intraspecific trait variation in southern European understorey species, with contrasting effects depending on the region. In their area of origin (central Italy), the species were mainly negatively affected in open stands with weak microclimatic buffering capacity. As recently suggested by Santi et al. (2025), supporting structurally complex and dense forests that can act as a refuge for shade‐adapted understorey specialists appears to be a suitable management choice in warm regions where the impact of increasing temperatures, solar radiation and drought is expected to increase in the future. When translocated beyond their northern range to an oceanic region, however, most species responded better in open forests, likely because of higher light availability and warmer conditions during the growing season. The flowering capacity shown by most species only 2 years after translocation to the north suggests a potential ability to persist beyond or close to their current range limits in a different macroclimatic context, also in terms of seasonal patterns of precipitation, air humidity and light regimes. Further translocation studies over a longer time span and accounting also for the effects of interspecific competition are necessary to better understand the effects of global warming on the life of Mediterranean understorey plants.

## Author Contributions

Federico Selvi, Pieter De Frenne and Cristina Gasperini conceived and designed the research. Cristina Gasperini performed the data analyses and wrote the manuscript with Federico Selvi. All authors performed fieldwork, collected data and contributed to the manuscript.

## Peer Review

The peer review history for this article is available at https://www.webofscience.com/api/gateway/wos/peer‐review/10.1111/ele.70184.

## Supporting information


**Appendix S1:** ele70184‐sup‐0001‐Supinfo1.docx.

**Appendix S2:** ele70184‐sup‐0002‐Supinfo2.docx.

## Data Availability

The data and codes that support the findings of this study are openly available from figshare at https://figshare.com/s/6a07fbddb449c35ce43b. We provide the R codes used for the analyses and producing the figures. All data required for meta‐analyses are provided https://doi.org/10.6084/m9.figshare.27004972.
