# Supplementary material for: Forest Density Drives Survival and Trait Variation in South European Understorey Species: A Continental‐Scale Translocation Experiment
Source: Ecol Lett. 2025 Aug 4;28(8):e70184. doi: 10.1111/ele.70184 (PMC12319884; doi:10.1111/ele.70184)
Supplement: Supplementary file 1 — Appendix S1: ele70184‐sup‐0001‐Supinfo1.docx. [file ELE-28-0-s001.docx]

**Supplementary materials A - Methods**

**Supplementary text A1** “Species description and plant material”

*Species description*. The eight understorey species used in the experiment are largely distributed in southern Europe (Euro+Med, 2006; Jaakko and Suominen, 1989; Meusel and Jager, 1992; Meusel et al., 1978, 1964) Four are typical of mountain woodlands dominated by beech (*Fagus sylvatica*) and four of lowland forests dominated by deciduous oaks (mainly *Quercus cerris* and *Q. pubescens*). Based on the Raunkiaer’s life-form system, five are geophytes with bulbs or rhizomes while three are hemicryptophytes, two of which with strong lateral spread by running stems (*Aegonychon purpurocaerulem, Glechoma hirsuta*), and one is a cespitose graminoid (*Luzula nivea*). According to the classification by Heinken *et al.* (2022), (Pignatti et al., 2017) and our expert evaluation (see also Gasperini *et al.* 2021) seven species are typical of closed forest (guild 1.1), while one (*Aegopodium podagraria*) is found in forest as well as open vegetation (guild 2.1). The studied species differ also in their phenological patterns, with some completing the production of new leaves, flowering and fruiting by mid-spring (i.e. *Anemone apennina*, *Cyclamen repandum*) while others completing these phases mostly in early summer (i.e. *Luzula nivea*, *Aegopodium podagraria*). Based on Ellenberg Indicator values for light, species were grouped in “more shade tolerant” (values 3-4: *Anemone trifolia, Luzula nivea, Geranium nodosum, Cyclamen repandum*) and “less shade tolerant” (values 5-6: *Aegopodium podagraria, Aegonychon purpureocaeruleum, Glechoma hirsuta, Anemone apennina*), see also Table A1. *Aegonychon purpureocaeruleum* and *Aegopodium podagraria* were collected at the edge of dense forests, all other species in the interior of dense forests.

*Plant Material*. In early spring 2020, small juvenile plants of the eight species above were collected from wild populations (one per species) at three native forest sites in central Italy, one at high altitude (Vallombrosa near Florence) and two at low altitude (Val di Sieve near Florence, Paganico near Grosseto; site details in Table 1). In total, ca. 800 individuals (ca. 100 per species) were collected with intact roots and transplanted in small peat pots (one plant per pot). The potted plants were placed in a nursery located in the same area of origin (i.e. the beech forest at the high-altitude site, the oak forest at one of the low-altitude sites) and kept there for 12 months to allow recovery and stabilisation after the transplant. Between February and March 2021, 64 healthy plants for each of the four mountain species and 60 for each of the lowland species were selected and used for the experiment, totalling 468 individuals (256 for the lowland species and 212 for the mountain species). The lower number of individuals in the mountain species group was due to the low survival rate of *Geranium nodosum* after the transplant (50%). For this reason, this species was used only in one of the two experimental sites (Italy, 32 plants).


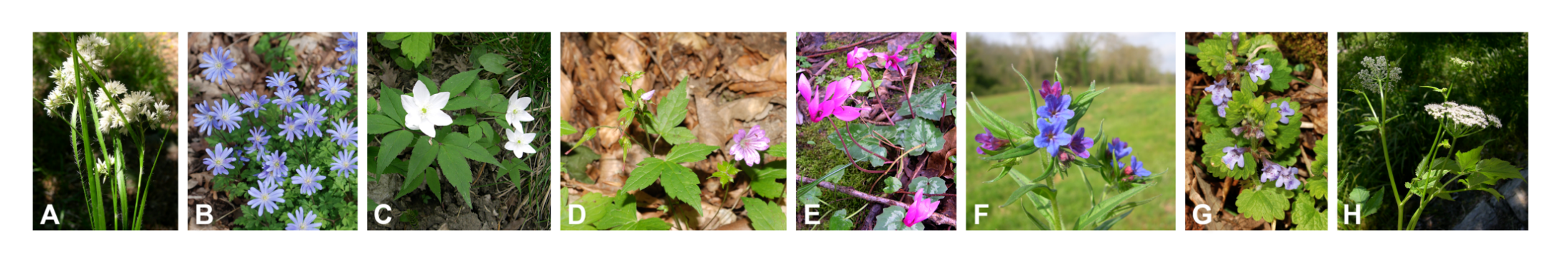


**Figure A1.** Study species: *Luzula nivea* (A), *Anemone apennina* (B), *Anemone trifolia* (C), *Geranium nodosum* (D), *Cyclamen repandum* (E), *Aegonychon purpurocaeruleum* (F), *Glechoma hirsuta* (G), *Aegopodium podagraria* (H).


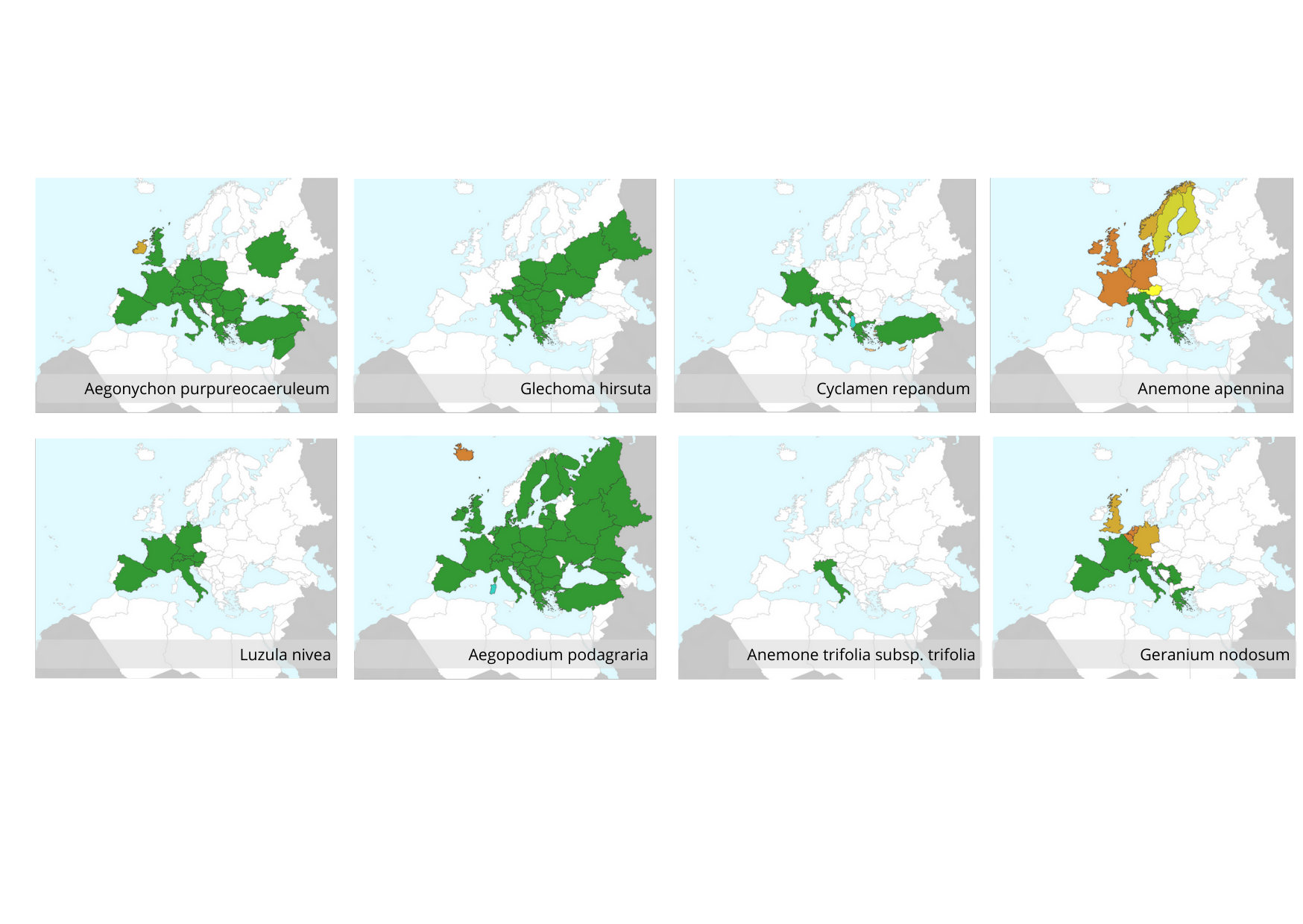


**Figure A2.** Approximated distribution maps of the eight study species following Euro+Med (2006) based on presence/absence in European countries. Green: native distribution, dark brown: naturalised alien, light brown: alien (status unknown), light green: casual alien, yellow: in large-scale cultivation.


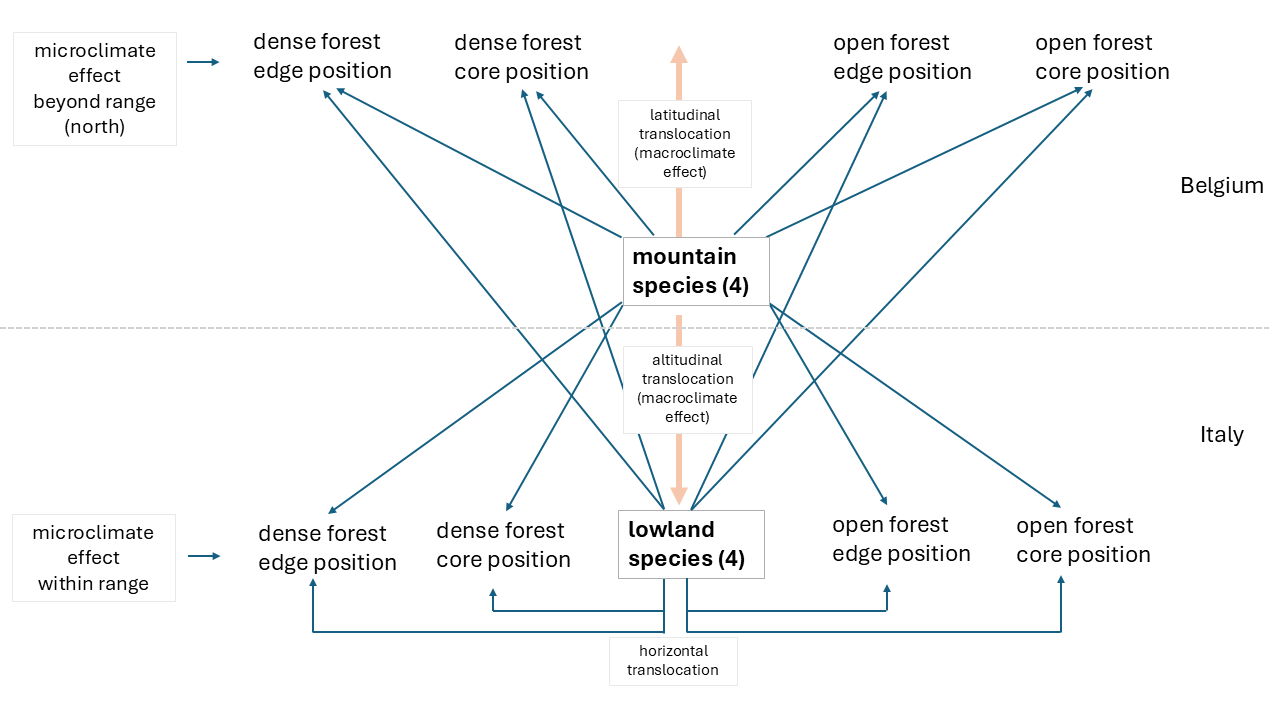


**Figure A3**. Schematic representation of the experimental design, showing the translocation of mountain and lowland species within and beyond range.


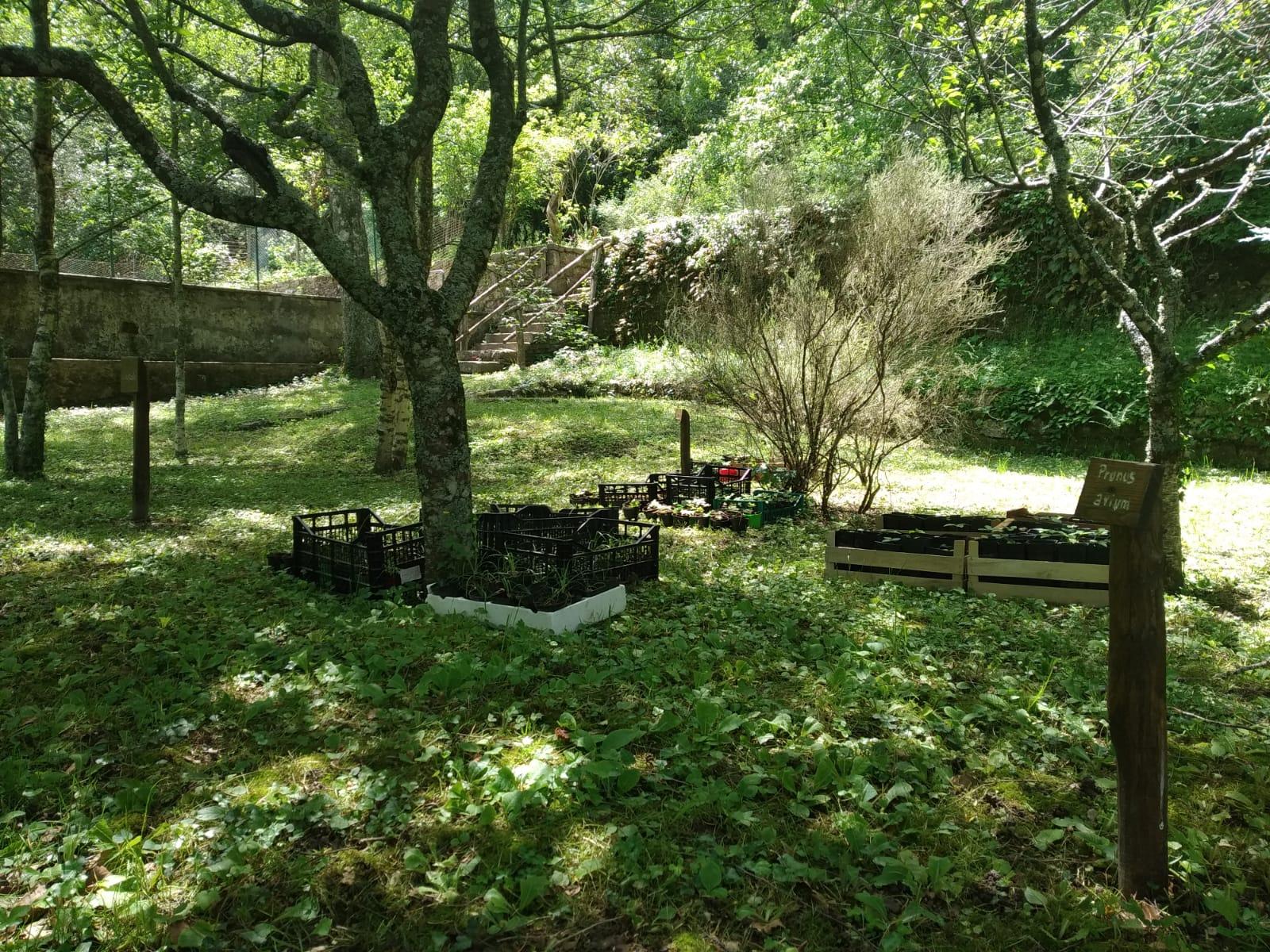


**Figure A4.** Nursery of mountain forest species. Plants were grown for ca. 1 year in conditions very similar to those of the collection site, before translocation.


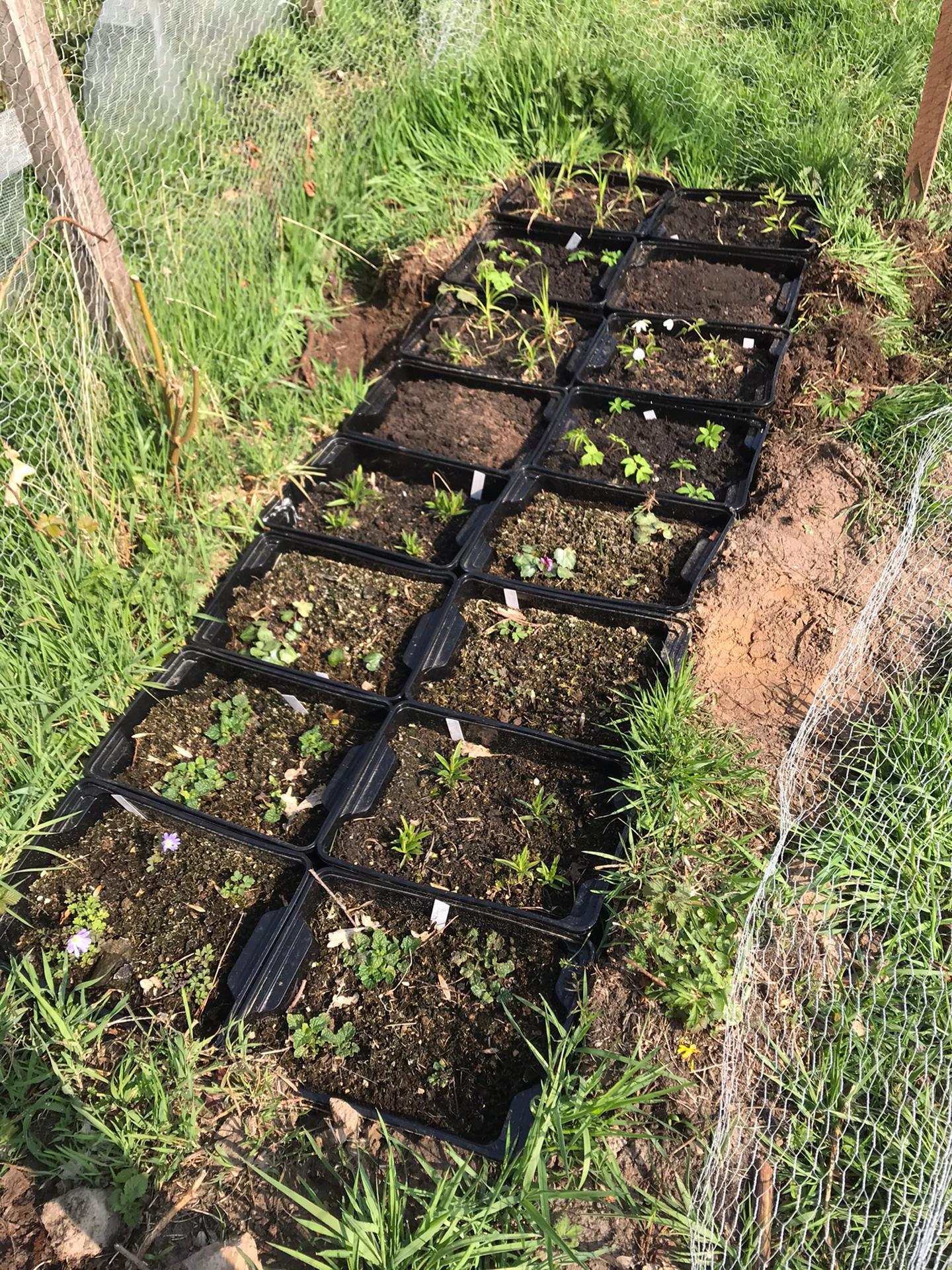


**Figure A5.** Experimental plot installed in the forest. The plastic boxes (volume 13.5 l) were all filled with a mixture of peat (95%) and expanded perlite (<5 %), partially buried in the forest soil and protected by a metallic fence against wild animals. A common potting soil was used to fill the boxes.

**Table A1**. Life-form, main distribution (chorotype) and ecological characteristics of the study species. Life-forms are G rhiz: rhizomatous geophyte, G bulb: bulbose geophyte, H caesp: caespitose hemicryptophyte, H scap: scapose hemicryptophyte, H rept: reptant hemicryptophyte); chorotype (Eurosib.: Eurosiberian, Orof.-Europ.: south European orophyte, Orof. SW-Europ: southwest European orophyte, SE-Europ.: southeast European, NW-Stenomedit.: northeast Steno-mediterranean); Ellenberg indicator values are after Pignatti *et al.* (2005). L: Light (scale 1-12, with 1 and 12 indicating lowest and highest shade tolerance, respectively); T: Temperature (scale 1-12, with 1 and 12 indicating highest and lowest cold tolerance, respectively); C: continentality (scale 1-9, with 1 and 9 indicating species of most oceanic and most continental climates, respectively); F: humidity (scale 1-12, with 1 indicating species of very arid sites and 12 the hydrophytes); R: soil reaction (scale 1-9, with 1 and 9 indicating highest acidophily and 9 highest basophily, respectively); N: soil nutrients (scale 1-9; with 1 and 9 indicating species with lowest and highest nutrient requirements; respectively), X: indifferent.

| **Species** | **Life form** | **Chorotype** | **L** | **T** | **C** | **F** | **R** | **N** |
| --- | --- | --- | --- | --- | --- | --- | --- | --- |
| **Mountain**  *Aegopodium podagraria* | G rhiz | Eurosib. | 5 | X | 4 | 6 | 7 | 8 |
| *Anemone trifolia* | G rhiz | Orof. S-Europ. | 4 | 5 | 5 | 6 | 6 | 7 |
| *Luzula nivea* | H caesp | Orof. SW-Europ. | 3 | 5 | 4 | 3 | 4 | 6 |
| *Geranium nodosum* | G rhiz | N-Medit.-Mont. | 4 | 4 | 4 | 6 | 6 | 6 |
|  |  |  |  |  |  |  |  |  |
| **Lowland**  *Aegonychon purpureocaeruleum* | H scap | Pontic | 5 | 7 | 6 | 4 | 8 | 4 |
| *Glechoma hirsuta* | H rept | SE-Europ. | 6 | 8 | 6 | 4 | 5 | 3 |
| *Anemone apennina* | G rhiz | SE-Europ. | 5 | 5 | 6 | 4 | 5 | 5 |
| *Cyclamen repandum* | G bulb | NW-Stenomedit. | 4 | 9 | 5 | 3 | X | 5 |

**Table** **A2.** Main characteristics of the experimental sites. Mean annual temperature (MAT) and mean total annual precipitation (MAP) from Worldclim 2 (average of years 1970-2000, resolution 9 km^2^). The open stands were characterized by a single tree layer, canopy cover < 70 % and maximum shrub cover of 45% and had been subject to more intense management (thinning) in the recent past, while the dense stands had canopy cover > 70%, two or three tree layers and shrub cover > 30%. The cover of tree and shrub layers were visually estimated in a circular plot with 9m radius around the experimental treatments. All the edges were south-facing, bordered by grassland or arable land.

| **Experiment**  **site** | **MAT**  **(°C)** | **MAP**  **(mm** **year^-1^)** | **Forest**  **structure** | **Latitude**  **(°)** | **Longitude**  **(°)** | **Position** | **Tree** **layer** **cover**  **(%)** | **Shrub** **layer** **cover**  **(%)** | **Dominant** **tree** **species** |
| --- | --- | --- | --- | --- | --- | --- | --- | --- | --- |
| Belgium  Ghent | 10.2 | 758 | Open | 50.9741 | 3.8046 | core | 60 | 30 | *Quercus robur, Castanea sativa* |
|  |  |  |  |  |  | edge | 20 | 15 | *Fraxinus excelsior, Ulmus glabra* |
|  |  |  | Dense | 50.9749 | 3.8043 | core | 98 | 60 | *Quercus robur, Acer pseudoplatanus,*  *Fraxinus excelsior* |
|  |  |  |  |  |  | edge | 90 | 30 | *Quercus robur, Fagus sylvatica, Castanea sativa, Acer pseudoplatanus* |
| Italy  Florence | 14 | 861 | Open | 43.6677 | 11.1394 | core | 60 | 43 | *Quercus pubescens, Quercus cerris,*  *Fraxinus ornus* |
|  |  |  |  |  |  | edge | 70 | 30 | *Quercus pubescens, Quercus cerris, Fraxinus ornus* |
|  |  |  | Dense | 43.6738 | 11.1422 | core | 70 | 40 | *Quercus pubescens, Quercus cerris,*  *Fraxinus ornus* |
|  |  |  |  |  |  | edge | 68 | 35 | *Quercus pubescens, Quercus cerris* |

**Table A3.** Minimum, mean and maximum temperatures recorded with Lascar thermologgers in the forest sites used for the translocation experiment. The Lascar thermologgers measured the temperature every 15 min in the air (15 cm height) (Wild et al., 2019) for two consecutive years (2020- 2021). Temperature sensors were protected from direct sunlight with white plastic shields. “Mean difference mountain and lowland species” refer to the difference between the mean annual temperature (MAT) between the site of collection of mountain/lowland species (macroclimatic temperatures) and the site of translocation (microclimatic temperature). Superscripts **^(1) (2)^** refer to different places of collection of lowland species (Group 1: *Anemone apennina* and *Cyclamen repandum*, Group 2: *Aegonychon purpurocaeruleum* and *Glechoma hirsuta*, see Table 1). Macroclimatic temperatures are based on data from Worldclim2 (most recent 30-years average from 1970 to 2000, resolution of 2.5 arcminutes (Fick and Hijmans, 2017). On average, mean temperatures were ca. 1 °C higher in the open than in the dense forest. Mean temperatures were slightly higher at the forest edge than at the core, though not consistently.

| **Temperature** | **Min.** | **Max.** | **Mean** | **Mean difference mountain species** | **Mean difference lowland species** ^(1)^ | **Mean difference lowland species** ^(2)^ |
| --- | --- | --- | --- | --- | --- | --- |
| BE- Dense edge | -8.9 | 35.6 | 11.2 | +1.6 | -2.1 | -3.5 |
| BE- Dense core | -8.6 | 34.8 | 10.9 | +1.3 | -2.5 | -3.8 |
| BE- Open edge | -9.4 | 46.6 | 10.9 | +1.4 | -2.5 | -3.7 |
| BE- Open core | -7.8 | 40.0 | 10.8 | +1.2 | -2.4 | -0.9 |
| IT- Dense edge | -6.9 | 37.0 | 14.2 | +4.6 | +0.8 | +0.5 |
| IT- Dense core | -6.4 | 39.0 | 13.7 | +4.2 | +0.3 | +0.9 |
| IT- Open edge | -6.3 | 40.5 | 14.7 | +5.2 | +1.3 | +0.1 |
| IT- Open core | -6.4 | 45.5 | 14.9 | +5.3 | +1.5 | +0.3 |

**Table A4.** Mean values of Photosynthetic Active Radiation (PAR, μm mol^-1^) in Italy and Belgium, measured at the different sites of plant translocation, e.g. in dense vs. open forest, and at the forest edge and the forest core. Measurements (18 per plot) were performed during the growing season (July) around midday (12:00 h–14:00 h) and under clean sky conditions.

|  | **Belgium** | | **Italy** |
| --- | --- | --- | --- |
| **Dense forest** | **11.73** | | **28.62** |
| Core | 12.94 | | 46.85 |
| Edge | 10.51 | | 10.38 |
| **Open forest** | **129.69** | | **176.64** |
| Core | 25.30 | | 157.22 |
| Edge | 234.09 | | 196.06 |
| **Overall mean** | **70.71** | | **102.63** |
|  | |  |  |
|  | |  |  |
|  | |  |  |
|  | |  |  |
|  | |  |  |
|  | |  |  |

**References**

Euro+Med, 2006. Euro+Med PlantBase - the information resource for Euro-Mediterranean plant diversity [WWW Document]. URL http://ww2.bgbm.org/EuroPlusMed/query.asp

Fick, S.E., Hijmans, R.J., 2017. WorldClim 2: new 1‐km spatial resolution climate surfaces for global land areas. Int. J. Climatol. 37, 4302–4315. https://doi.org/10.1002/joc.5086

Gasperini, C., Carrari, E., Govaert, S., Meeussen, C., De Pauw, K., Plue, J., Sanczuk, P., Vanneste, T., Vangansbeke, P., Jacopetti, G., De Frenne, P., Selvi, F., 2021. Edge effects on the realised soil seed bank along microclimatic gradients in temperate European forests. Sci. Total Environ. 798, 149373. https://doi.org/10.1016/j.scitotenv.2021.149373

Heinken, T., Diekmann, M., Liira, J., Orczewska, A., Schmidt, M., Brunet, J., Chytrý, M., Chabrerie, O., Decocq, G., De Frenne, P., Dřevojan, P., Dzwonko, Z., Ewald, J., Feilberg, J., Graae, B.J., Grytnes, J., Hermy, M., Kriebitzsch, W., Laiviņš, M., Lenoir, J., Lindmo, S., Marage, D., Marozas, V., Niemeyer, T., Paal, J., Pyšek, P., Roosaluste, E., Sádlo, J., Schaminée, J.H.J., Tyler, T., Verheyen, K., Wulf, M., Vanneste, T., 2022. The European forest plant species list (EuForPlant): Concept and applications. J. Veg. Sci. 33. https://doi.org/10.1111/jvs.13132

Jaakko, J., Suominen, J., 1989. Atlas Flora Europaeae. Distribution of vascular plants in Europe. Cambridge University Press.

Meusel, H.., Jager, E., 1992. Vergleichende Chorologie der zentraleuropa ̈ischen Flora – Karten. Veb Gustav Fischer Verlag, Jena III.

Meusel, H., Jager, E., Rauschert, S., Weinert, E., 1978. Vergleichende Chorologie der zentraleuropa ̈ischen Flora –Karten. Veb Gustav Fischer Verlag, Jena II.

Meusel, H., Jager, E., Weinert, E., 1964. Vergleichende Chorologie der zentraleuropa ̈ischen Flora – Karten. Veb Gustav Fischer Verlag, Jena I.

Pignatti, S., Guarino, R., La Rosa, M., 2017. Flora d’Italia, 2nd Edition. Edagricole-New Business Media.

Pignatti, S., Menegoni, P., Pietrosanti, S., 2005. Braun-Blanquetia. Bioindicator values of vascular plants of the Flora of Italy. Università degli studi di Camerino, Camerino.

Wild, J., Kopecký, M., Macek, M., Šanda, M., Jankovec, J., Haase, T., 2019. Climate at ecologically relevant scales: A new temperature and soil moisture logger for long-term microclimate measurement. Agric. For. Meteorol. 268, 40–47. https://doi.org/10.1016/j.agrformet.2018.12.018
