# Supplementary material for: Forest Density Drives Survival and Trait Variation in South European Understorey Species: A Continental‐Scale Translocation Experiment
Source: Ecol Lett. 2025 Aug 4;28(8):e70184. doi: 10.1111/ele.70184 (PMC12319884; doi:10.1111/ele.70184)
Supplement: Supplementary file 2 — Appendix S2: ele70184‐sup‐0002‐Supinfo2.docx. [file ELE-28-0-s002.docx]

**Supplementary materials B - Results**

**Table B1.** Marginal R^2^ and conditional R^2^ (Nakagawa and Schielzeth, 2013) of linear mixed models (lmer) and generalized linear mixed models (glmer) for each species for the four investigated variables: plant cover, height, leaf number and SLA. Marginal R^2^ and conditional R^2^ represent the proportion of variation explained respectively by fixed factors and both random and fixed factors*.*

| **Taxa** |  | **Cover** | **Plant height** | **Leaf number** | **SLA** |
| --- | --- | --- | --- | --- | --- |
| *Anemone apennina* | R^2^ marginal | 0.11 | 0.08 | 0.25 | 0.34 |
|  | R^2^ conditional | 0.57 | 0.71 | 0.91 | 0.48 |
| *Cyclamen repandum* | R^2^ marginal | 0.27 | 0.05 | 0.22 | 0.43 |
|  | R^2^ conditional | 0.38 | 0.83 | 0.60 | 0.62 |
| *Aegonychon purpureocaeruleum* | R^2^ marginal | 0.20 | 0.11 | 0.30 | 0.26 |
|  | R^2^ conditional | 0.21 | 0.12 | 0.89 | 0.26 |
| *Glechoma hirsuta* | R^2^ marginal | 0.18 | 0.12 | 0.20 | 0.06 |
|  | R^2^ conditional | 0.21 | 0.15 | 0.96 | 0.06 |
| *Anemone trifolia* | R^2^ marginal | 0.08 | 0.05 | 0.08 | 0.34 |
|  | R^2^ conditional | 0.32 | 0.37 | 0.25 | 0.57 |
| *Aegopodium podagraria* | R^2^ marginal | 0.19 | 0.21 | 0.26 | 0.19 |
|  | R^2^ conditional | 0.20 | 0.21 | 0.57 | 0.35 |
| *Luzula nivea* | R^2^ marginal | 0.19 | 0.14 | 0.21 | 0.06 |
|  | R^2^ conditional | 0.57 | 0.65 | 0.98 | 0.25 |
| *Geranium nodosum* | R^2^ marginal | 0.03 | 0.06 | 0.06 | 0.02 |
|  | R^2^ conditional | 0.18 | 0.66 | 0.77 | 0.63 |

**Table B2.** Means ± standard deviations of the response variables per species grouped by mountain (M) and lowland (L) species.

|  | **ITALY** | | | | **BELGIUM** | | | |
| --- | --- | --- | --- | --- | --- | --- | --- | --- |
| **Trait** | **DENSE FOREST** | | **OPEN FOREST** | | **DENSE FOREST** | | **OPEN FOREST** | |
|  | **Edge** | **Core** | **Edge** | **Core** | **Edge** | **Core** | **Edge** | **Core** |
| **Survival (%) M** | 87.09 | 87.09 | 67.74 | 64.51 | 22.73 | 52.17 | 73.91 | 45.45 |
| **L** | 81.25 | 78.13 | 50 | 56.25 | 28.13 | 43.75 | 75 | 12.5 |
| **Plant height (cm) M** | 16.19 ± 7.62 | 19.35 ± 8.24 | 16.63 ± 9.32 | 14.69 ± 6.79 | 12.09 ± 4.01 | 17.26 ± 6.18 | 27.1 ± 16.23 | 16.67 ± 12.79 |
| **L** | 25.44 ± 24.66 | 24.15 ± 28.94 | 21.40 ± 26.16 | 19.74 ± 19.92 | 10.37 ± 11.16 | 11.44 ± 10.39 | 39.33 ± 42.73 | 23.59 ± 23.00 |
| **Leaves (n) M** | 8.15 ± 7.05 | 12.33 ± 13.19 | 8.93 ± 10.63 | 10.24 ± 14.93 | 9.66 ± 9.81 | 11.27 ± 17.09 | 55.2 ± 86.31 | 24 ± 63.16 |
| **L** | 18.68 ± 16.49 | 16.29 ± 14.71 | 16.01 ± 18.13 | 18.57 ± 13.43 | 8.85 ± 6.83 | 12.46 ± 11.26 | 50.86 ± 82.05 | 21.32 ± 16.76 |
| **Cover (%) M** | 20.52 ± 34.17 | 19.66 ± 12.92 | 14.23 ± 7.10 | 13.74 ± 6.99 | 12.19 ± 5.29 | 17.17 ± 7.64 | 24.14 ± 10.78 | 12.81 ± 10.70 |
| **L** | 17.01 ± 8.94 | 13.65 ± 6.23 | 13.97 ± 8.09 | 12.70 ± 5.66 | 9.68 ± 5.97 | 11.87 ± 7.50 | 30.87 ± 38.02 | 13.49 ± 11.18 |
| **Leaf area (mm^2^) M** | 35113.94 ± 35360.23 | 30757.33 ± 26958.71 | 23758.92 ± 20328.34 | 23242.37 ± 35882.68 | 16232.88 ± 22381.09 | 25374.48 ± 26161.45 | 22626.75 ± 22966.77 | 7268.71 ± 5987.22 |
| **L** | 8116.11 ± 6551.06 | 9586.54 ± 7299.35 | 6644.14 ± 4793.18 | 5203.87 ± 4046.49 | 4824.86 ± 2349.25 | 9697.38 ± 11992.08 | 8823.97 ± 9327.33 | 8126.9 ± 9218.16 |
| **Leaf dry weight (mg) M** | 100.97 ± 156.90 | 84.59 ± 122.07 | 71.11 ± 80.29 | 83.65 ± 133.56 | 47.81 ± 50.42 | 70.77 ± 90.38 | 91.05 ± 154.92 | 37.60 ± 39.54 |
| **L** | 28 ± 31.85 | 27.65 ± 35.24 | 19.39 ± 18.23 | 21.55 ± 20.08 | 27.68 ± 23.54 | 45.81 ± 48.60 | 25.34 ± 33.45 | 20.49 ± 22.97 |
| **SLA (mm^2^/mg) M** | 220.39 ± 99.07 | 229.58 ±135.43 | 217.51 ±128.82 | 181.36 ± 85.10 | 160.34 ± 81.26 | 198.21 ± 97.59 | 106.03 ± 45.83 | 128.97 ± 90.72 |
| **L** | 240.29 ±101.66 | 258.22 ±116.55 | 229.94 ±103.32 | 175.37 ± 61.92 | 187.61 ± 191.21 | 205.83 ± 158.06 | 145.57 ± 83.15 | 202.37 ± 121.23 |
| **Flowering (%) M** | 22.58 | 35.48 | 19.35 | 41.94 | 9.09 | 26.09 | 50 | 18.18 |
| **L** | 31.25 | 28.13 | 34.38 | 18.75 | 6.25 | 31.25 | 37.5 | 3.13 |

**Table B3.** Means of the response variables of the eight study species translocated to dense and open forests of the two regions.

| **Forest**  **type** | **Region** | **Species** | **Height**  **(cm)** | **Leaves**  **(n)** | **Cover**  **(%)** | **Leaf area**  **(mm^2^)** | **Leaf dry weight**  **(mg)** | **SLA**  **(mm^2^/mg)** | **Survival**  **(%)** | **Flowering**  **(%)** |
| --- | --- | --- | --- | --- | --- | --- | --- | --- | --- | --- |
| Dense | BE | Aeg.pod | 20.6 | 2 | 17.0 | 48076.4 | 61.1 | 212.0 | 21 | 0 |
| Open | BE | Aeg.pod | 23.5 | 7 | 24.1 | 45596.8 | 141.6 | 135.0 | 50 | 36 |
| Dense | IT | Aeg.pod | 21.4 | 5 | 20.5 | 58317.2 | 132.9 | 218.6 | 100 | 6 |
| Open | IT | Aeg.pod | 13.7 | 4 | 13.5 | 30400.2 | 53.3 | 199.8 | 81 | 0 |
| Dense | BE | Aeg.pur | 30.8 | 17 | 16.5 | 6273.3 | 5.1 | 320.1 | 0 | 0 |
| Open | BE | Aeg.pur | 72.8 | 53 | 51.1 | 7312.0 | 19.4 | 171.5 | 56 | 0 |
| Dense | IT | Aeg.pur | 73.8 | 43 | 24.4 | 4717.0 | 8.9 | 252.9 | 63 | 0 |
| Open | IT | Aeg.pur | 54.7 | 29 | 15.0 | 3834.9 | 8.1 | 194.4 | 31 | 0 |
| Dense | BE | Ane.app | 9.0 | 13 | 13.8 | 6106.9 | 35.3 | 124.9 | 81 | 56 |
| Open | BE | Ane.app | 4.5 | 4 | 5.4 | 3718.0 | 10.8 | 86.6 | 19 | 0 |
| Dense | IT | Ane.app | 8.9 | 6 | 12.0 | 7801.0 | 37.1 | 163.6 | 100 | 50 |
| Open | IT | Ane.app | 8.5 | 19 | 12.4 | 3139.1 | 14.9 | 146.5 | 81 | 50 |
| Dense | BE | Ane.tri | 11.5 | 4 | 10.7 | 13510.3 | 31.9 | 237.5 | 69 | 50 |
| Open | BE | Ane.tri | 9.4 | 3 | 7.1 | 10514.4 | 36.4 | 125.4 | 53 | 7 |
| Dense | IT | Ane.tri | 9.4 | 3 | 9.4 | 12526.2 | 35.7 | 259.5 | 94 | 31 |
| Open | IT | Ane.tri | 11.2 | 4 | 11.2 | 17437.2 | 44.4 | 219.6 | 63 | 31 |
| Dense | BE | Cyc.rep | 5.6 | 3 | 5.5 | 13064.0 | 46.7 | 119.2 | 63 | 19 |
| Open | BE | Cyc.rep | 4.2 | 3 | 4.0 | 5785.0 | 18.9 | 93.2 | 50 | 38 |
| Dense | IT | Cyc.rep | 8.3 | 6 | 12.1 | 15544.0 | 58.0 | 203.4 | 100 | 63 |
| Open | IT | Cyc.rep | 7.0 | 4 | 8.3 | 9702.4 | 29.9 | 183.6 | 100 | 56 |
| Dense | IT | Ger.nod | 20.4 | 11 | 35.6 | 40568.9 | 106.2 | 244.0 | 88 | 50 |
| Open | IT | Ger.nod | 17.7 | 8 | 15.8 | 24904.3 | 47.0 | 248.9 | 56 | 50 |
| Dense | BE | Gle.hir | 27.9 | 3 | 15.8 | 27527.0 | 4.8 | 611.4 | 0 | 0 |
| Open | BE | Gle.hir | 72.0 | 99 | 55.9 | 21752.5 | 31.3 | 307.8 | 44 | 44 |
| Dense | IT | Gle.hir | 39.6 | 18 | 19.7 | 12471.1 | 13.1 | 419.9 | 56 | 6 |
| Open | IT | Gle.hir | 40.3 | 9 | 13.4 | 8772.6 | 7.9 | 309.8 | 0 | 0 |
| Dense | BE | Luz.niv | 12.0 | 26 | 16.8 | 3041.6 | 13.9 | 80.9 | 19 | 0 |
| Open | BE | Luz.niv | 28.8 | 99 | 27.3 | 3382.4 | 26.2 | 86.6 | 75 | 69 |
| Dense | IT | Luz.niv | 18.9 | 22 | 15.3 | 5795.3 | 26.0 | 116.3 | 64 | 29 |
| Open | IT | Luz.niv | 22.3 | 21 | 14.3 | 4999.1 | 32.5 | 81.3 | 64 | 43 |

**Table B4**. Mean values of the response variables for the eight study species translocated in edge and core position in the two regions.

| **Edge vs core** | **Region** | **Species** | **Height**  **(cm)** | **Leaves**  **(n)** | **Cover**  **(%)** | **Leaf area**  **(mm^2^)** | **Leaf dry weight**  **(mg)** | **SLA**  **(mm^2^/mg)** | **Survival**  **(%)** | **Flowering**  **(%)** |
| --- | --- | --- | --- | --- | --- | --- | --- | --- | --- | --- |
| Core | BE | Aeg.pod | 19.0 | 1.8 | 15.6 | 48076.4 | 81.4 | 212.0 | 21 | 0 |
| Core | IT | Aeg.pod | 18.4 | 5.1 | 16.4 | 44621.7 | 64.1 | 212.8 | 81 | 0 |
| Edge | BE | Aeg.pod | 25.0 | 7.2 | 25.7 | 45596.8 | 106.2 | 135.0 | 50 | 36 |
| Edge | IT | Aeg.pod | 17.9 | 4.5 | 18.5 | 47683.9 | 121.7 | 209.3 | 100 | 6 |
| Core | BE | Aeg.pur | 46.5 | 26.8 | 23.7 | 8411.8 | 12.5 | 239.5 | 13 | 0 |
| Core | IT | Aeg.pur | 61.7 | 37.8 | 16.5 | 4783.5 | 9.9 | 226.0 | 56 | 0 |
| Edge | BE | Aeg.pur | 63.9 | 47.0 | 47.0 | 5703.0 | 12.1 | 213.9 | 44 | 0 |
| Edge | IT | Aeg.pur | 69.9 | 35.6 | 24.5 | 3757.3 | 6.9 | 225.8 | 38 | 0 |
| Core | BE | Ane.app | 10.1 | 15.9 | 13.8 | 5785.7 | 18.8 | 141.9 | 56 | 44 |
| Core | IT | Ane.app | 7.9 | 10.1 | 11.2 | 4864.1 | 20.6 | 154.4 | 81 | 38 |
| Edge | BE | Ane.app | 5.8 | 5.4 | 9.7 | 5352.5 | 28.7 | 96.2 | 50 | 13 |
| Edge | IT | Ane.app | 9.5 | 13.3 | 13.0 | 6076.1 | 30.9 | 155.8 | 100 | 63 |
| Core | BE | Ane.tri | 10.9 | 3.5 | 8.9 | 13155.2 | 45.4 | 213.2 | 87 | 47 |
| Core | IT | Ane.tri | 11.2 | 3.7 | 11.2 | 14993.1 | 43.5 | 250.4 | 88 | 56 |
| Edge | BE | Ane.tri | 9.9 | 3.2 | 9.3 | 10695.1 | 23.7 | 151.1 | 40 | 13 |
| Edge | IT | Ane.tri | 9.2 | 2.3 | 9.2 | 14416.9 | 35.9 | 225.9 | 69 | 6 |
| Core | BE | Cyc.rep | 5.4 | 3.3 | 6.8 | 13968.0 | 68.6 | 119.0 | 38 | 19 |
| Core | IT | Cyc.rep | 7.9 | 6.4 | 12.0 | 14811.5 | 50.7 | 189.5 | 100 | 56 |
| Edge | BE | Cyc.rep | 4.9 | 2.4 | 3.9 | 6156.0 | 15.6 | 98.7 | 75 | 38 |
| Edge | IT | Cyc.rep | 7.5 | 4.0 | 9.2 | 11783.0 | 40.4 | 202.1 | 100 | 63 |
| Core | IT | Ger.nod | 21.6 | 10.5 | 21.5 | 31760.6 | 83.1 | 234.4 | 69 | 56 |
| Edge | IT | Ger.nod | 17.2 | 8.3 | 32.6 | 35460.6 | 68.9 | 258.9 | 75 | 44 |
| Core | BE | Gle.hir | 35.5 | 13.6 | 20.3 | 22560.6 | 13.8 | 466.9 | 6 | 6 |
| Core | IT | Gle.hir | 38.7 | 11.7 | 13.6 | 11435.4 | 11.0 | 348.3 | 31 | 0 |
| Edge | BE | Gle.hir | 80.1 | 120.9 | 76.4 | 23109.9 | 22.3 | 262.6 | 38 | 38 |
| Edge | IT | Gle.hir | 41.1 | 17.0 | 20.5 | 10817.1 | 10.1 | 411.5 | 25 | 6 |
| Core | BE | Luz.niv | 17.5 | 44.3 | 20.1 | 3442.9 | 20.6 | 98.8 | 38 | 19 |
| Core | IT | Luz.niv | 20.7 | 24.2 | 15.4 | 4842.5 | 25.5 | 93.1 | 56 | 43 |
| Edge | BE | Luz.niv | 25.9 | 91.7 | 25.6 | 3028.8 | 19.6 | 68.7 | 56 | 50 |
| Edge | IT | Luz.niv | 20.5 | 18.3 | 14.3 | 5941.5 | 33.0 | 105.2 | 56 | 29 |


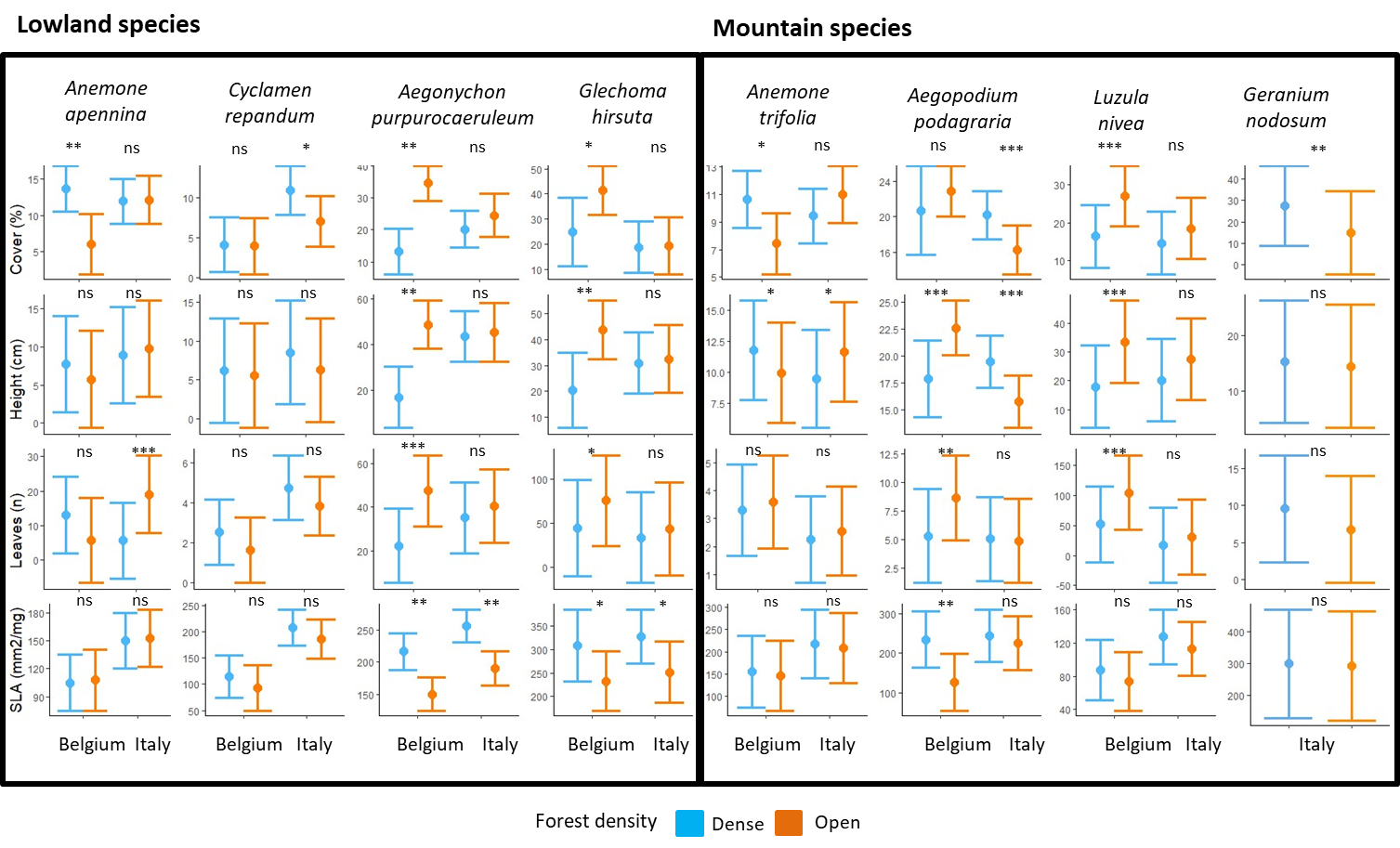


**Figure B1**. Interactions between forest structure (open vs dense) and region (Italy vs Belgium), based on lmer and glmer, for each species for plant cover (scaled), height (scaled), leaf number and SLA (scaled). When no interaction between forest type and region was included in the final model we showed the significance level referred to the variable “forest type” (referred to both regions).


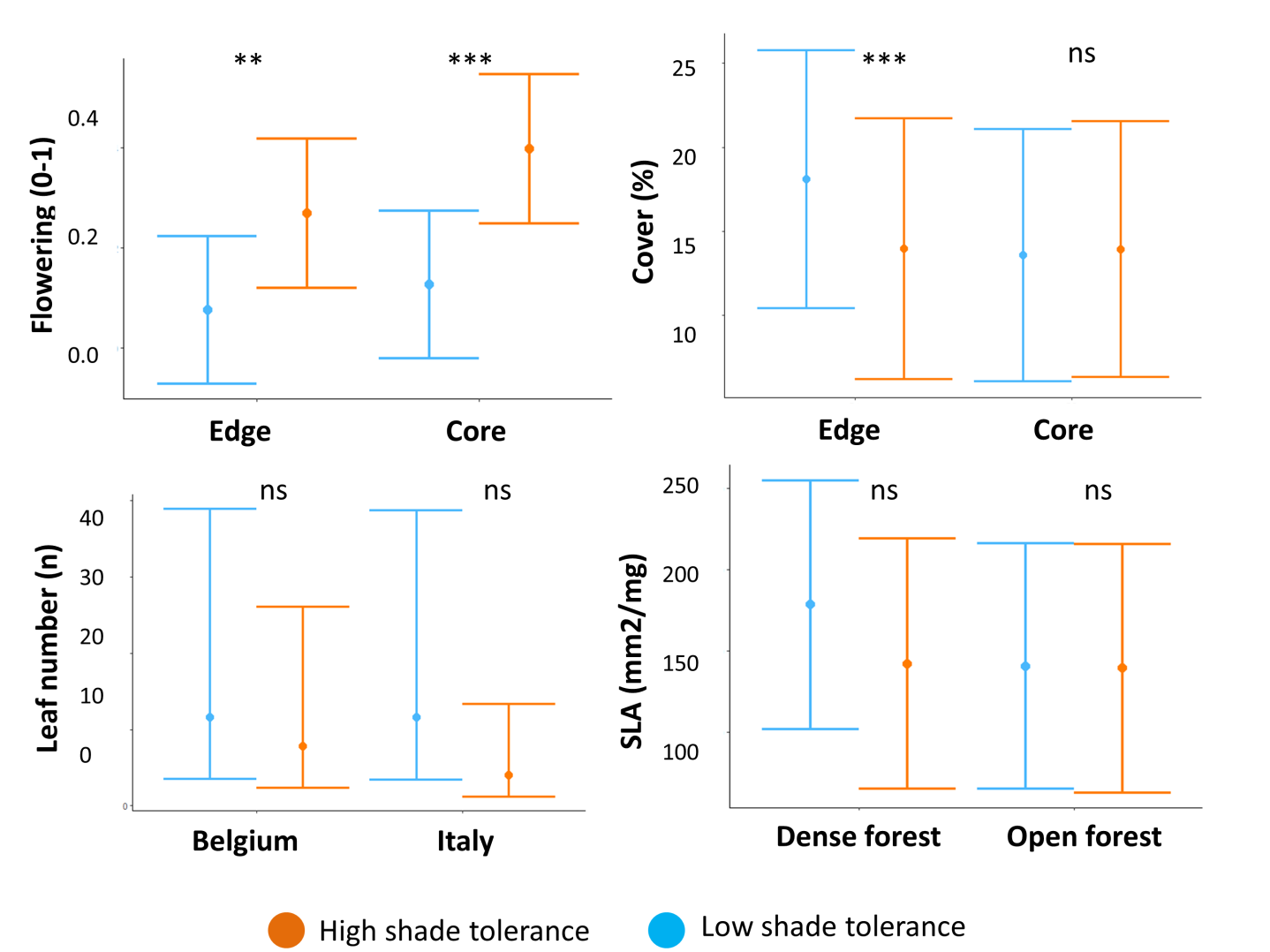


**Figure B2.** Significant interactions from generalized and linear mixed effects models (GLMm and LMMs) including the factor “shade tolerance”. Shade tolerance in the study species was based on Ellenberg indicator values for light (L) requirements; it was lower in *Anemone trifolia, Luzula nivea, Geranium nodosum* and *Cyclamen repandum* (L=3-4) and higher in *Aegopodium podagraria, Aegonychon purpureocaeruleum, Glechoma hirsuta, Anemone apennina* (L=5-6); see also Table A1.

**References**

Nakagawa, S., Schielzeth, H., 2013. A general and simple method for obtaining R2 from generalized linear mixed-effects models. Methods Ecol. Evol. 4, 133–142. https://doi.org/10.1111/j.2041-210x.2012.00261.x
